# Explainable detection: a transformer-based language modeling approach for Bengali news title classification with comparative explainability analysis using ML and DL

**DOI:** 10.3389/frai.2025.1537432

**Published:** 2025-11-06

**Authors:** Md. Julkar Naeen, Sourav Kumar Das, Sakib Alam Jisan, Sharun Akter Khushbu, Noyon Chandra Saha, Ohidujjaman

**Affiliations:** 1 Department of Computer Science and Engineering, Daffodil International University, Dhaka, Bangladesh; 2 Department of Computer Science and Engineering, United International University, Dhaka, Bangladesh

**Keywords:** transformer model, long short-term memory, Bengali news titles, classification, LIME, explainable AI, machine learning, deep learning

## Abstract

Classifying scattered Bengali text is the primary focus of this study, with an emphasis on explainability in Natural Language Processing (NLP) for low-resource languages. We employed supervised Machine Learning (ML) models as a baseline and compared their performance with Long Short-Term Memory (LSTM) networks from the deep learning domain. Subsequently, we implemented transformer models designed for sequential learning. To prepare the dataset, we collected recent Bengali news articles online and performed extensive feature engineering. Given the inherent noise in Bengali datasets, significant preprocessing was required. Among the models tested, XLM-RoBERTa Base achieved the highest accuracy 0.91. Furthermore, we integrated explainable AI techniques to interpret the model’s predictions, enhancing transparency and fostering trust in the classification outcomes. Additionally, we employed LIME (Local Interpretable Model-agnostic Explanations) to identify key features and the most weighted words responsible for classifying news titles, which validated the accuracy of Bengali news classification results. This study underscores the potential of deep learning models in advancing text classification for the Bengali language and emphasizes the critical role of explainability in AI-driven solutions.

## 1 Introduction

Information is the most significant asset in the modern world. People utilize various platforms to access information, with newspapers being one of the most common and accessible sources. Newspapers offer a wealth of information on diverse topics at an affordable price, making knowledge accessible to everyone. They enrich readers’ understanding and provide insight into domestic and international current events. Newspaper has become quite easy in today’s world. Humans can easily comprehend news headlines and their underlying meanings due to their familiarity with the language and context. However, this task poses significant challenges for machines, particularly when processing text in Bengali. In the Bengali language, many words have multiple meanings that vary depending on their context and usage, making it difficult for machines to interpret their intended meaning accurately. Additionally, some news headlines are lengthy, further complicating the extraction of semantic information from these complex sentences. To address this issue, it is essential to train models capable of understanding the contextual meaning of sentences. Transformer-based models are particularly well-suited for this task, as they leverage a deeply bidirectional architecture, enabling them to capture the contextual relationships within a sentence. Consequently, transformers are a robust choice for deriving the semantic meaning of words and sentences, offering a significant advantage in tasks involving natural language understanding. Bengali, the national language of Bangladesh, is spoken by approximately 300 million people worldwide, drawing significant attention in the field of Natural Language Processing (NLP) (Hossain et al., 2020a). Recent research on Bengali text has been extensive. In response, we aimed to innovate in Bengali news article classification. We gathered raw data from various newspapers, balanced it for better performance, processed it, and applied machine learning and deep learning models, along with explainable AI. Our goal was to classify articles based on their titles. Our model can predict the category from headlines of varying lengths, effectively handling Bengali words with multiple meanings depending on context. This capability enhances our research’s accuracy. If successful, our study could spark further interest among NLP researchers. Classifying Bengali newspaper articles is challenging due to certain linguistic complexities. However, overcoming these challenges could yield promising results, as deep learning and NLP provide optimal solutions for text classification problems.

Bengali text classification is quite popular nowadays. In the newspaper, people give different opinions about national, international, politics, sports, etc. Our work is related to identifying different classes from titles. Sentiment analysis is a prominent aspect of NLP research, emphasizing the importance of identifying words that convey positive and negative meanings (Roy et al., 2023). Additionally, fake news, prevalent even in newspapers, can mislead people and obscure the truth (Fouad et al., 2022). Most algorithms struggle with plain text, making word embedding understanding essential (Wadud et al., 2022). The Transformer is a recent neural network model, well-supported for English but lacking resources for Bengali text classification (Alam et al., 2020). Fake news detection is also prevalent in other languages (Das et al., 2023a). Despite the significant resource gap for the Bengali language in NLP, some researchers have managed to categorize Bengali sentences into different forms (Das et al., 2023b). Sentiment analysis remains crucial, successfully detecting emotions in sentences (Bhowmik et al., 2021). For emotions like anger, disgust, fear, joy, sadness, and surprise in Bengali, researchers have proposed an interesting transformer-based method (Sourav et al., 2022). Cyberbullying is a common issue today, prompting NLP researchers to explore prevention methods (Ahmed et al., 2021). Malicious activities targeting government security also pose a significant problem (Aslam et al., 2022). LIME is an effective tool for explaining black-box machine learning models in various fields (Venkatsubramaniam and Baruah, 2022).

Our findings reveal a substantial body of research on the Bengali language. However, comparatively limited work focuses on classifying and identifying the semantic meaning of words or sentences within the contextually rich and often ambiguous structure of Bengali. Many Bengali sentences carry multiple meanings depending on their situational and contextual usage, posing significant challenges for machines in accurately discerning their underlying meaning. This research seeks to address these challenges. Several machine learning and deep learning models, including a Long Short-Term Memory (LSTM) network, were employed in this study. While these models demonstrated strong performance in classifying the dataset, they fell short in capturing words and sentences’ deeper, contextual semantics. LSTM and traditional machine learning models struggle to understand nuanced meanings that depend heavily on context. In contrast, transformer-based models named XLM-RoBERTa base (Conneau, 2019) and Multilingual BERT (Kenton and Toutanova, 2019) outperformed these approaches. Due to their deeply bidirectional architecture and superior capability to learn contextual and semantic nuances, transformers are more effective in understanding the true meaning of sentences. This makes them a more suitable choice for processing the Bengali language, where semantic ambiguity is prevalent.

This study aims to explore the following research questions:

1. How can Bengali news headlines be accurately classified despite the contextual ambiguity and multiple meanings of Bengali words?
2. To what extent can transformer-based models, such as XLM-RoBERTa and Multilingual BERT, outperform traditional machine learning and deep learning models (e.g., LSTM) in classifying Bengali newspaper headlines?
3. Can Explainable AI techniques, such as LIME, effectively reveal which parts of Bengali headlines influence model decisions, thereby improving interpretability?

We contributed to our dataset by creating our own properly annotated data on news lines collected from various sources. We also contributed to the comparison of conventional approaches, deep learning, and transformer models. We customized the squeeze and attention blocks in the transformer model to achieve smaller weights, enhancing efficiency and producing a low-loss graph. After evaluating the model’s performance, we utilized Explainable AI (XAI) to gain deeper insights into Bengali word interpretations within the hidden layers. This helped identify which words contributed more to the model and which were processed for the next iteration. We applied the LIME technique, which revealed that headline-related words carried higher weights during text learning.

## 2 Related work

Until recently, there was limited research on the Bengali language in fields like machine learning, deep learning, and NLP. There has been some work done in this area, but it is still not very advanced and is not very common. Several studies have employed various BERT models, but none have incorporated Explainable AI (XAI) techniques. Despite using BERT, some report lower accuracy than our model. Our approach, integrating both BERT and XAI, yields improved performance. In earlier studies, models like Random Forest, Multinomial Naive Bayes, and LSTM were used in similar ways. BERT made these methods more effective and efficient.

Maisha et al. (2021) did sentiment analysis of Bengali newspapers by implementing supervised machine learning algorithms. Some techniques were combined for the class. From all the six models, Random Forest provided the best accuracy of 99%. Bhowmik et al. (2022) did the sentiment analysis with an extended lexicon dictionary and deep learning, and the highest accuracy was in the BERT-LSTM model. As well, sentiment analysis was done by Hassan et al. (2022) for Bengali conversation, and support vector machine (SVM) gave the best accuracy, which is 85.59%. Prottasha et al. (2022) also did sentiment analysis on behalf of BERT-based supervised fine-tuning. Word embedding techniques like Word2Vec, GloVe, and fastText are used. CNN-BiLSTM provided the highest accuracy of 94.15%. After that, Keya et al. (2022) also used BERT to classify fake news and created the AugFake-BERT model. To implement the model, more than 50,000 data points are used. However, the proposed model provided an accuracy of 92.45%, and all the other scores are utilized to evaluate the performance. By using BERT, Kowsher et al. (2022) created the Bengali-BERT model for language understanding and transfer learning. Bengali-BERT performed better than the other models, with 97.03% accuracy. Then, Hossain et al. (2020b) categorized Bengali news headlines with deep learning models. For classification, two models are used: LSTM and GRU. Both models provided almost the same accuracy but with a little difference. GRU shows the highest accuracy of 87.74%. Hossain et al. (2020c) classified Bengali news using dissimilar machine learning-based baseline approaches and deep learning models. A total of 3,000 data were used to implement the models. SVC, LSVC, Random Forest, Linear Regression, Naive Bayes, CNN, and BiLSTM models are applied for the classification, and the highest accuracy of 93.43% came from the CNN model. Dhar and Morshed (2022) analyzed Bengali crime news categorization with the help of machine learning models. From different newspapers, a total of 3,500 data were collected for the implementation. After training the data, the proposed model shows a test accuracy of 87%. Subsequently, Yeasmin et al. (2021) proposed a topic about Bengali news classification using ML and Neural Network models. A total of two datasets were used for the process. One of the datasets was collected from the Bengali newspapers, and the other was collected from Kaggle. One neutral network model provided the highest accuracy of 92.63% for dataset 1. For dataset 2, a neural network model again offered the highest accuracy of 95.50%. Applying CNN, RNN, and other deep learning models might give better outcomes. Zhang (2021) also researched the application of deep learning in news text classification on different datasets. However, the models were CNN, MLP, LSTM, and some hybrid models were used, and one hybrid model outnumbered all other models and displayed 94.82% accuracy. Similarly, Ramdhani et al. (2020) used convolutional neural networks (CNN) to classify Indonesian news. CNN has the best accuracy of 90.74%, with a value loss of 29.05%. Saigal and Khanna (2020) applied SVM-based classifiers to classify the category of news. Some ML and hybrid deep learning models were used in the research, and a hybrid model named LS-TWSVM showed the highest accuracy, which was 98.21% on a specific dataset named the Reuters dataset. The dataset was collected from UCI News datasets like Reuters and 20 Newsgroups. After that, Mridha et al. (2021) created the L-Boost model, which can identify abusive words from social media posts in Bengali. ML model AdaBoost and DL model LSTM are combined with a transformer (BERT). The model reached 95.11% accuracy, which is the highest among all the ML and DL models. Sen et al. (2022) processed Bengali natural language for comprehensive analysis. Classical, machine learning, and deep learning applied on the study. A total of 75 BNLP research papers were studied and categorized into 11 categories for the research. Here we examined recent works that employed similar approaches, including machine learning and deep learning models such as Multinomial Naive Bayes, SVC, LSTM, as well as techniques like TF-IDF, BERT, and others.

At present, a lot of research is going on in the Bengali language. Using machine learning and deep learning models, many studies are ongoing. Just like that, Hasan et al. (2023a) classified Bengali newspaper headlines by using LSTM, Bi-LSTM, and Bi-GRU models. Almost 10,000 data points were classified into six categories to achieve the expected result. From those three deep learning models, the Bi-LSTM provides the highest train and test accuracy of 97.96 and 77.91%, respectively. Al Mahmud et al. (2023) similarly proposed an approach to classifying Bengali news by using machine learning and deep learning models. Applied models are SVC, Random Forest, LSVC, LSTM, and GRU. The approach technique provided an accuracy of 95.45%, which is the highest among all the algorithms. Hussain et al. (2023) also did some comparison analysis of Bengali news article classification using some ML models. TF-IDF and count vectorizer were used for the feature extraction process. SVM and LR algorithms were applied, and SVM provided the highest accuracy of 84%. There were 20 categories, and 12.5 K labeled news articles were used. Adding more categories and applying more ML and deep learning might give a more optimal result. After that, Mahmud et al. (2023) evaluated news by using Natural Language Processing (NLP) and Human Expert Opinion. A total of three NLP models were applied for training and testing. The Bengali-Bertbase model provided the highest testing accuracy of 84.99%, and it increased after the 9th parameter, where it achieved 93.80% of testing accuracy. Then, Hasan et al. (2023b) did sentiment analysis and natural language processing (NLP) using transformers for Russia-Ukraine war-based comments in Bengali. The applied models for the analysis are mBERT, Distil-mBERT, BengaliBERT, XML-R(base), XML-R (large), and Bi-LSTM. The BengaliBERT model performed best and provided an accuracy of 86% with a 0.82 F1 score. Ahmed et al. (2023) also did Bengali sentiment analysis. E-commerce sentiment classification is done by using transformer-based and transfer learning models. A total of three models are applied for the analysis: LSTM, GRU, and BengaliBERT. For binary classification and multiclass classification, the highest accuracy was 94.5 and 88.78% in BengaliBERT, respectively. After that, Tareq et al. (2023) worked with cross-linguistic contextual understanding on Bengali-English code-mixed sentiment analysis. Several machine learning and deep learning models were applied with word embedding models to analyze the data. Among them, XGBoost with the code-mixed Fasttext model gained the best F1 score of 0.87. Haque et al. (2023) researched Bengali social media comments for multiclass sentiment classification by using machine learning models. 42,036 Facebook comments trained with features like TF-IDF, CV, and Word2Sequence are applied to several machine learning and deep learning models. Among all the models, CLSTM with the Word2Sequence model performed better than all with an accuracy of 87.80%.

In the part of Explainable AI (XAI), there are few studies done. If we see Kawakura et al. (2022) utilized Explainable AI (XAI) techniques based on SHAP, LIME, and LightGBM to analyze agricultural worker datasets. These systems use sensors that are attached to worker’s bodies to gather information about how they move in farming. Data scientists use Python programs on devices to look at farm movements and find patterns that can help train farmers. After that, Dieber and Kirrane (2020) also worked with Explainable AI to investigate the use of LIME. This study evaluates different mathematical procedures to examine how LIME can be used to understand decisions in fields like healthcare and self-driving cars, testing the comprehensibility of LIME’s results. We looked over XAI papers that we used in our work, and LIME is a similar method that we used in our study.

## 3 Data and methodology

This section provides details about our dataset and the models used in the research. In section 3, we disclosed several contribution details through subsections. In 3.1, mention of the Dataset description. 3.2 describes how the data was collected. Subsection 3.3 is the steps of data pre-processing. In 3.5, ML models are summarized. Finally, 3.6 LSTM describes its layers.

**Figure 1:**
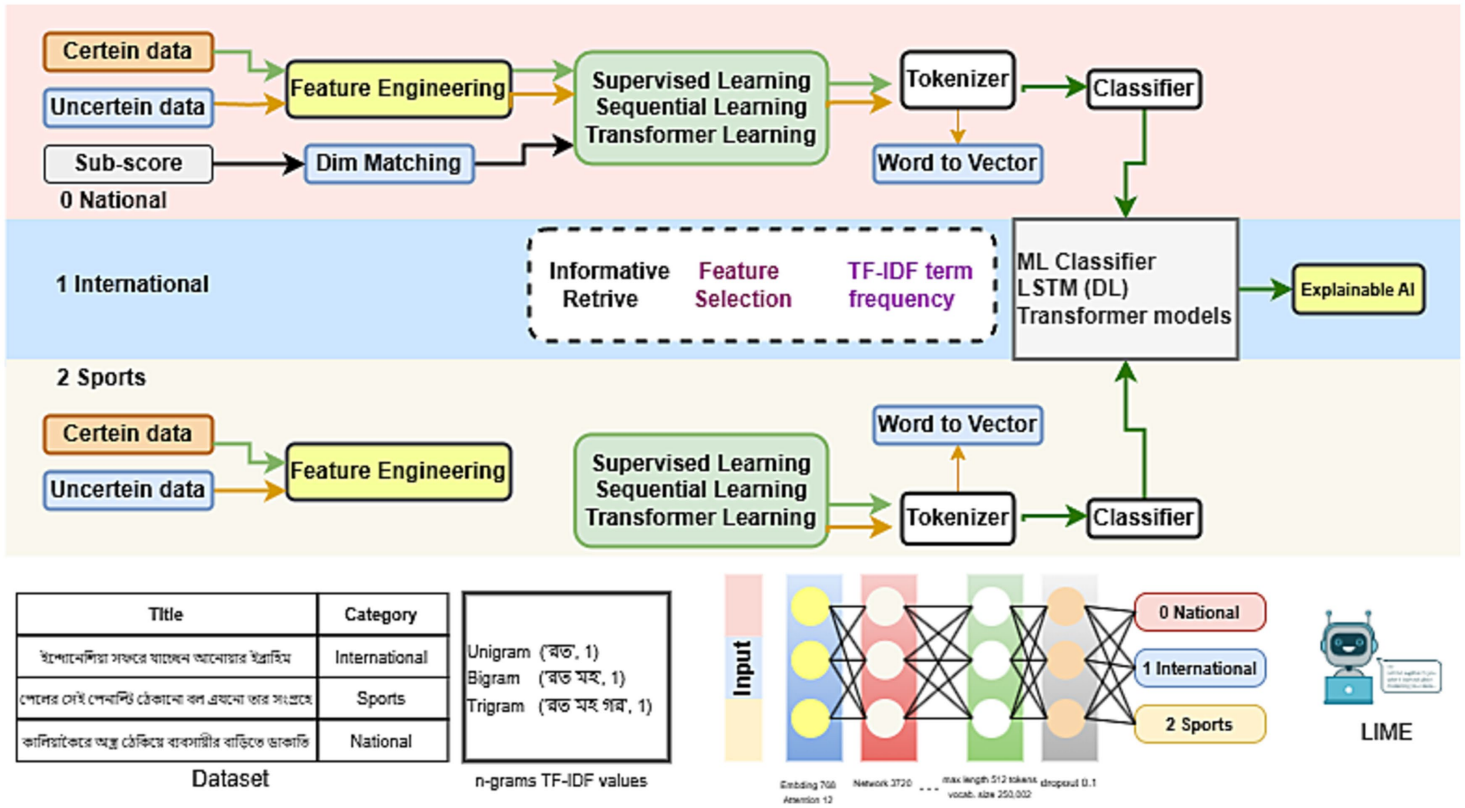
Architecture of the methodology for Bengali Title Classification.

Figure 1 illustrates the workflow of our study, including how we collected data and applied feature engineering. It also highlights the number of classes and types of data present in the dataset. After selecting the necessary features, we utilized ML classifiers, LSTM, and Transformer models, along with Explainable AI techniques. Additionally, the research incorporated the use of N-grams and TF-IDF for feature extraction and analysis.

### 3.1 Dataset collection

We prepared a raw dataset by ourselves. Bengali news articles are published on the newspaper websites of various newspapers as e-papers (Timeline, n.d.). The dataset is prepared manually from these four newspapers: Prothom Alo, Ittefaq, Jugantor, and Kaler Konto. We have uploaded the dataset on Mendeley, and it is publicly available (Julkar Naeen and Sourav Kumar Das, 2024).

We ensured proper data annotation by accurately labeling the text data with clear and consistent guidelines. This approach minimized ambiguity and maintained the integrity of the dataset, facilitating effective training and evaluation of the text classification model. Regular quality checks were performed to verify annotation accuracy, ensuring reliability. This meticulous process enhanced the model’s ability to learn and deliver precise predictions.

### 3.2 Dataset description

**Figure 2:**
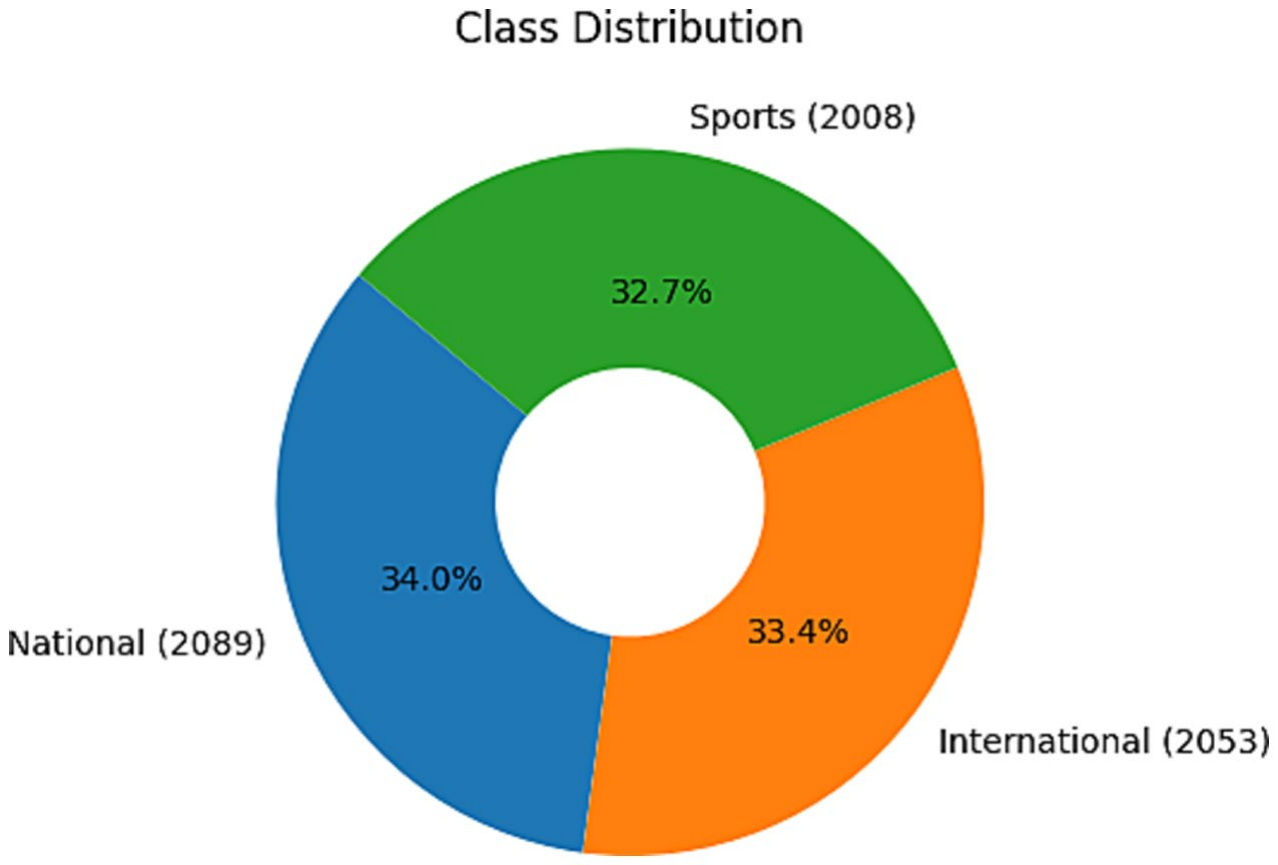
Class distribution and quantity visualization of three classes.

The dataset is about news articles from newspapers. The dataset has four attributes: title, publisher, newspaper name, and publication date. A total of 6,150 titles are taken in the dataset. Figure 2 provides visualization of the dataset’s quantity and distribution based on the three classes. 2089 titles are classified as national, 2008 are classified as sports, and 2053 as international.

National: Political, social, economic, and cultural news pertaining to events in Bangladesh.

International: News about events and developments taking place outside Bangladesh, but typically of regional or international significance.

Sports: Updates on local and global sporting competitions, teams, and players’ performances.

Table 1 shows a sample of the dataset where the title column has the titles and the category column has the category of the titles. An additional column shows the translated English of titles.

**Table 1:** Sample of the total dataset of Bengali news titles.

Title	Category
ইন্দোনেশিয়া সফরে যাচ্ছেন আনোয়ার ইব্রাহিম Anwar Ibrahim is visiting Indonesia	International
পেলের সেই পেনাল্টি ঠেকানো বল এখনো তার সংগ্রহে Pele’s penalty save ball is still in his collection	Sports
কালিয়াকৈরে অস্ত্র ঠেকিয়ে ব্যবসায়ীর বাড়িতে ডাকাতি Armed robbery at businessman’s house in Kaliakore	National

We aimed to gather approximately 2,000 samples for each class since we know that transfer learning models are data-hungry and would perform well with additional data. While reviewing related research, we noted that some research works made use of much less data. Trying to do better than these works, we consciously tried to gather more data than these benchmarks. After collecting some 2,000 samples per class—6,150 data points in all—we could see a definite improvement in model performance. Even though 6,150 samples are not particularly large, it was enough for our experimental aims. Also, the data was hand-labeled, so the process of collection was laborious and difficult.

### 3.3 Dataset pre-processing

**Figure 3:**
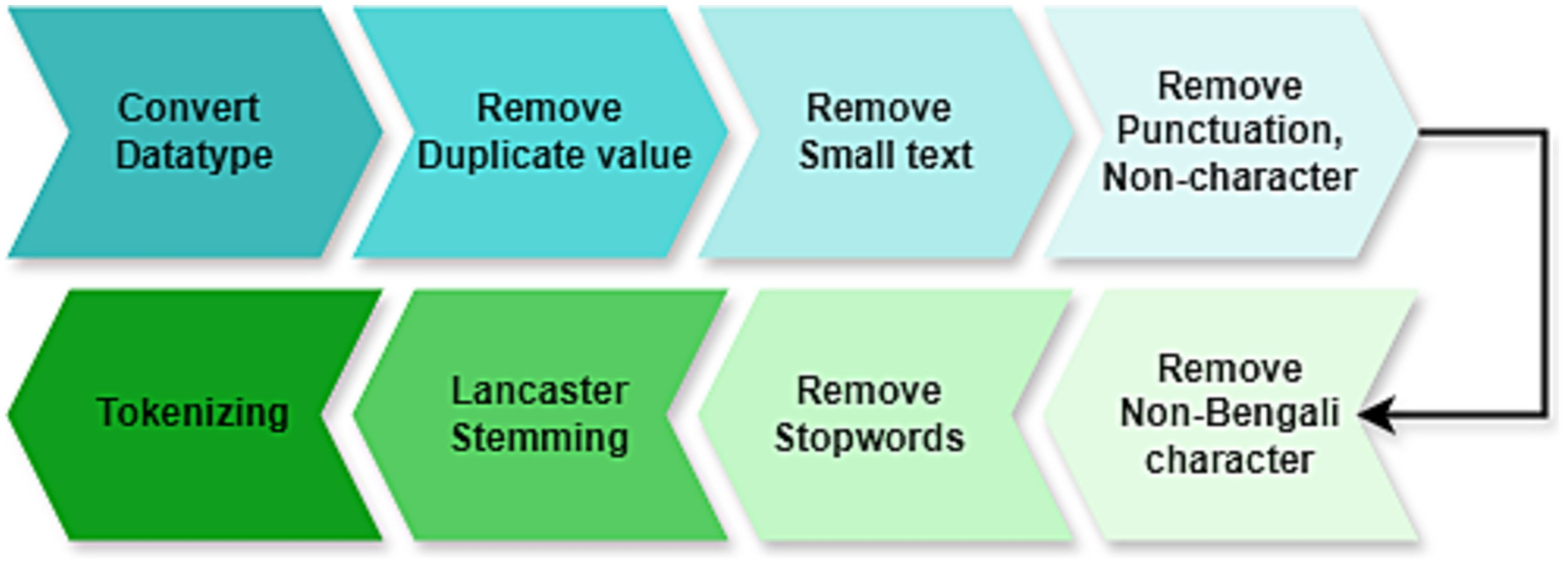
Preprocessing of Bengal news titles using the necessary steps.

Processing the data is the crucial part for cleaning the data and making ready for training ML and DL models. Figure 3 presents the steps followed in data pre-processing. This pre-processing step includes data cleaning by removing unnecessary items from the dataset, and then removing stopwords, tokenizers, stemming, null value handling, removing duplicate values, small texts that have no meaning and punctuations, and non-Bengali characters, then removed stopwords, then used Lancaster stemming and tokenized the dataset. For example, “ভাষাশিক্ষক মিথিলা”(Language teacher Mithila). This sentence will not help models identify their category. Steps of pre-processing the data the following order.

a. Convert Data Types: First of all, the total dataset is converted to a string type so that models can learn easily. Provides consistency for text models (e.g., BERT, LSTM) that require string inputs.
b. Remove Duplicate Row: In case there were any duplicate values, these steps removed all the duplicate values or data if there existed any in the dataset. Removes redundancy, avoiding model bias to overrepresented samples.
c. Remove Small Text: A title that has a length of less than four words seems not to make sense or is not understandable. For this situation, a small text of titles that consists of fewer than four words was removed. Brief messages tend to miss contextual information, damaging model.
d. Remove Punctuation, Link, Emoji (No Character): Punctuation marks, links, and non-character items create problems for the machine in learning. So non-characters and punctuation are removed. It reduces tokenization noise, particularly for subword models like BERT. Stripping punctuation is particularly necessary for agglutinative languages like Bengali, where suffixes are meaningful but extraneous symbols are not.

Some of the punctuations are ‘,’, ‘!’, ‘?’, ‘।’.

e. Remove Non-Bengali Character: Since it’s a Bengali dataset and the total research is on Bengali text, having non-Bengali characters in the data means an anomaly. So, all the non-Bengali characters are removed. It trains the model’s capacity on Bengali language patterns, free from interference due to mixed-language noise. Essential for monolingual tasks like sentiment analysis or topic classification.

Remove Stopwords: Stopwords are usually those words that do not have significant meaning in Bengali (Luhn, 1958), so these cannot be taken as a tokenizer. Thus, these are noisy data. Moreover, stopwords ain’t verbs or tenses and so generate ambiguity. Table 2 shows some of the Bengali stopwords. While processing Bengali text, these stopwords are removed for models to understand Bengali text and perform better. This step reduces noise. These noises are created because of several uses of these words. Table 3 has three columns showing the results of removing the stopwords from the sentences.

f. Stemming: In NLP, stemming means bringing words to their root form. Using this technique, words are reduced to their base form. In this research, Bengali text is produced for training models so that machines can understand it more accurately. Table 4 shows the sentences before and after stemming. An additional column was added to the previous tables. This research is conducted using Lancaster stemming (Paice, 1990) on the dataset. In Lancaster stemming, there is no change in the sentences, which are shown in Table 4. Stemming covers morphological variation in Bengali (i.e., verb conjugations, plural markers), grouping semantically similar words together. Improves model efficiency but over-stems in some cases.
g. Tokenizer: Tokenizing refers to splitting the sentences into raw units such as words (Salton, 1983). It helps to transform unprocessed text data into a more structured. Allows out-of-vocabulary words via Byte-Pair Encoding (BPE), crucial for compound words in Bengali.

**Table 2:** Some stop words in Bengali.

Bengali stopwords	Verbatim
এই	This
এবং	And
একটি	A/An
তো	That
তাহলে	Then
কিন্তু	But
কবে	When
যা	Which
কোনো	Any
কিছু	Some

**Table 3:** Samples of stopwords removed from the dataset.

Title	After removing stopwords
এক বার জুতোর ফিতে বেঁধেই ১ কোটি ২৩ লাখ 1 crore 23 lakhs for tying shoelaces once	এক জুতোর ফিতে বেঁধেই ১ ২৩ লাখ
তেল উত্তোলনে চীনা প্রতিষ্ঠানের সঙ্গে তালেবানের চুক্তি Taliban deal with Chinese companies to extract oil	তেল উত্তোলনে চীনা প্রতিষ্ঠানের তালেবানের চুক্তি
নতুন স্বপ্ন নিয়ে ২০২৩ সালকে বরণ বিশ্ববাসীর People of the world welcome the year 2023 with new dreams	স্বপ্ন নিয়ে ২০২৩ সালকে বরণ বিশ্ববাসীর

**Table 4:** Sample of the titles before and after stemming.

Title	After lancaster stemming
২১ বছর ভারত ছেলেকে ফেরত পেলেন মা বাবা 21-year-old Indian parents got their son back	২১ বছর ভারত ছেলেকে ফেরত পেলেন মা বাবা
স্বাধীনতা দিবসের প্রাক্কালে যুক্তরাষ্ট্রের বন্দুক হামলায় নিহত ১৫ 15 killed in gun attack in the US on the eve of Independence Day	স্বাধীনতা দিবসের প্রাক্কালে যুক্তরাষ্ট্রের বন্দুক হামলায় নিহত ১৫
মাকে বাঁচাতে ভাইয়ের হাতে বোন খুন Sister killed by brother to save mother	মাকে বাঁচাতে ভাইয়ের হাতে বোন খুন

Sample Sentence: “আইনজীবী হত্যা মামলায় ইমরানখানকে সুপ্রিম কোর্টে তলব.”

Interpreted Sample: “Imran Khan summoned to Supreme Court in lawyer murder case.”

Token Words: [“আইনজীবী (lawyer),” “হত্যা (murder),” “মামলায় (in case),” “ইমরানখানকে (Imran Khan),” “সুপ্রিম (Supreme),” “কোর্টে (Court),” “তলব (summoned)”].

### 3.4 Data summary

The summary produces the length of the words and the length of the characters. Also, the total number of sentences for different classes, the total number of words for each class, the total number of unique words for each class, and their number. These details help us to select the appropriate models for the research, as well as the NLP techniques.

**Figure 4:**
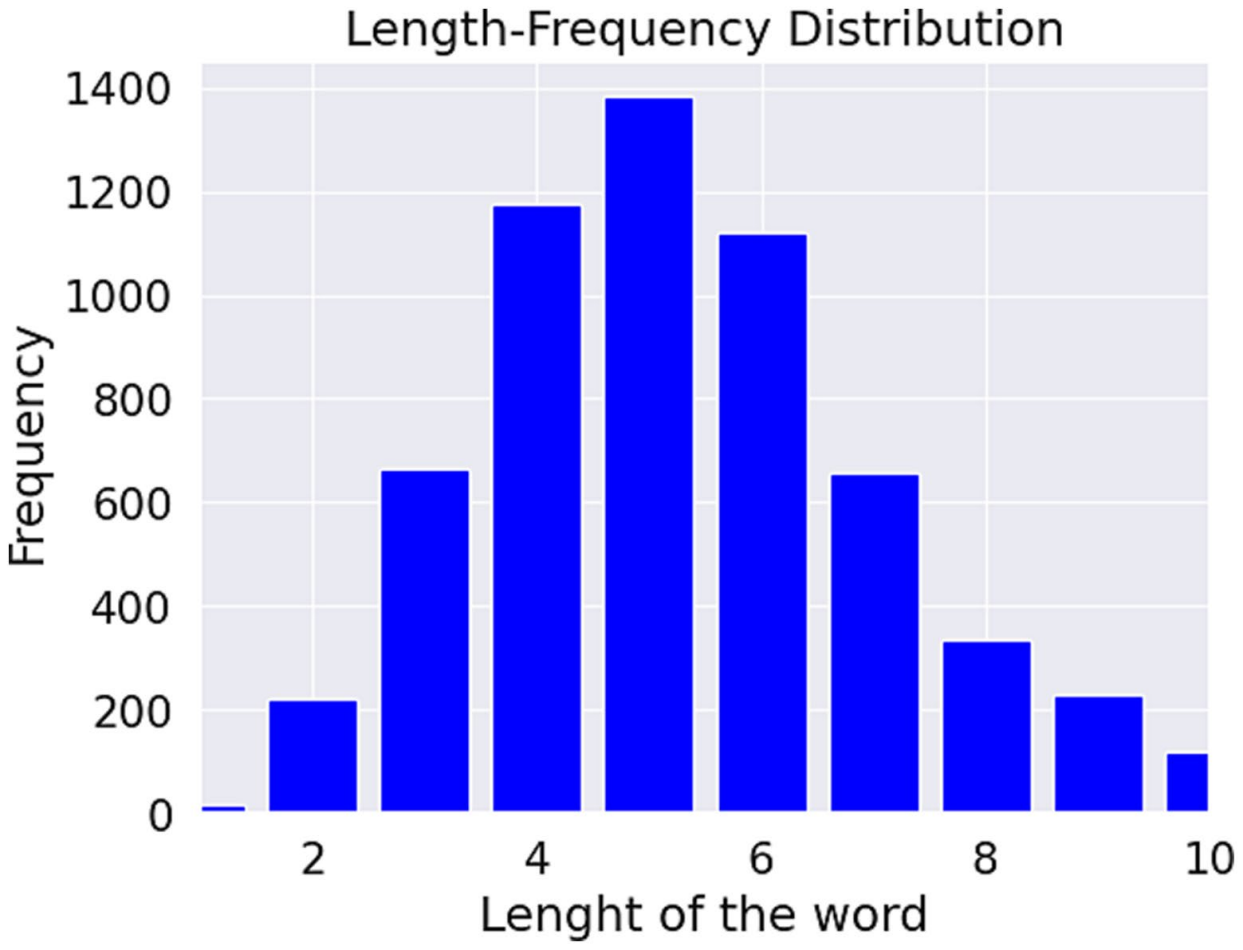
Length of the word frequency distribution.

**Figure 5:**
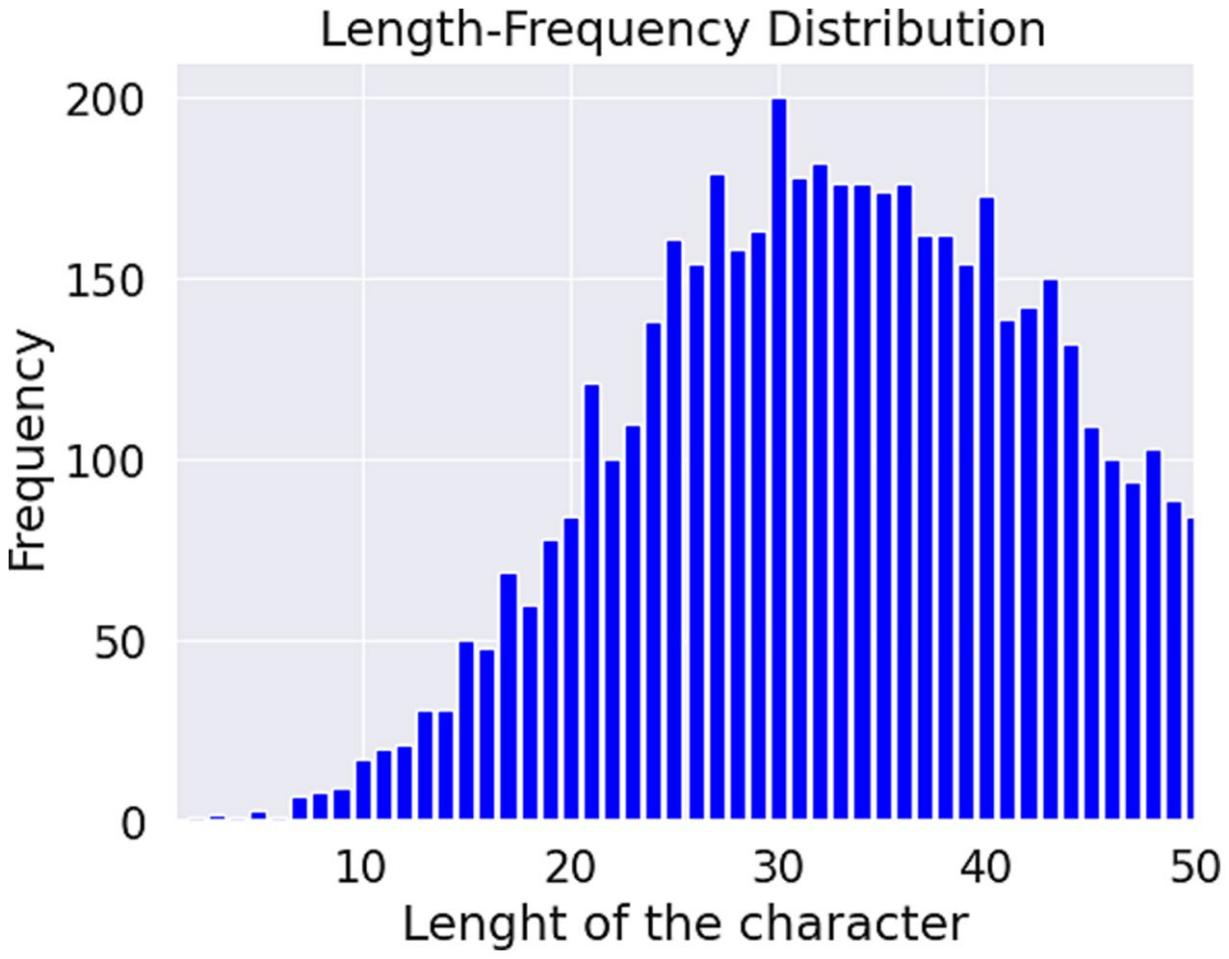
Length of the character frequency distribution.

Figures 4, 5. show the length-frequency distribution of words and characters, respectively.

**Figure 6:**
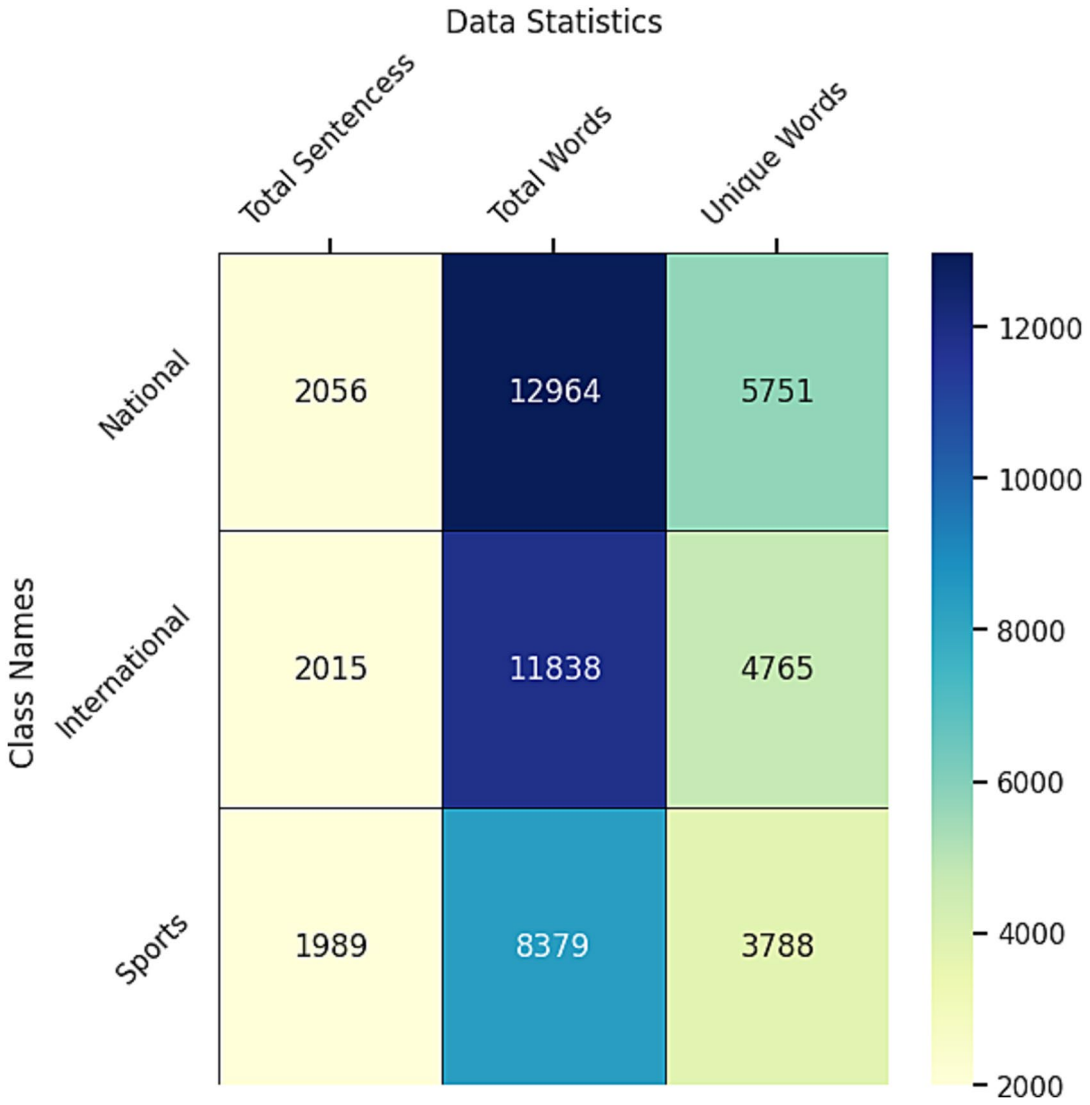
Data statistics after processing the dataset.

Moreover, Figure 6 shows that 2056 sentences are classified as national, 2015 sentences are in the international category, and 1989 sentences are sports-related in the dataset after the preprocessing.

**Figure 7:**
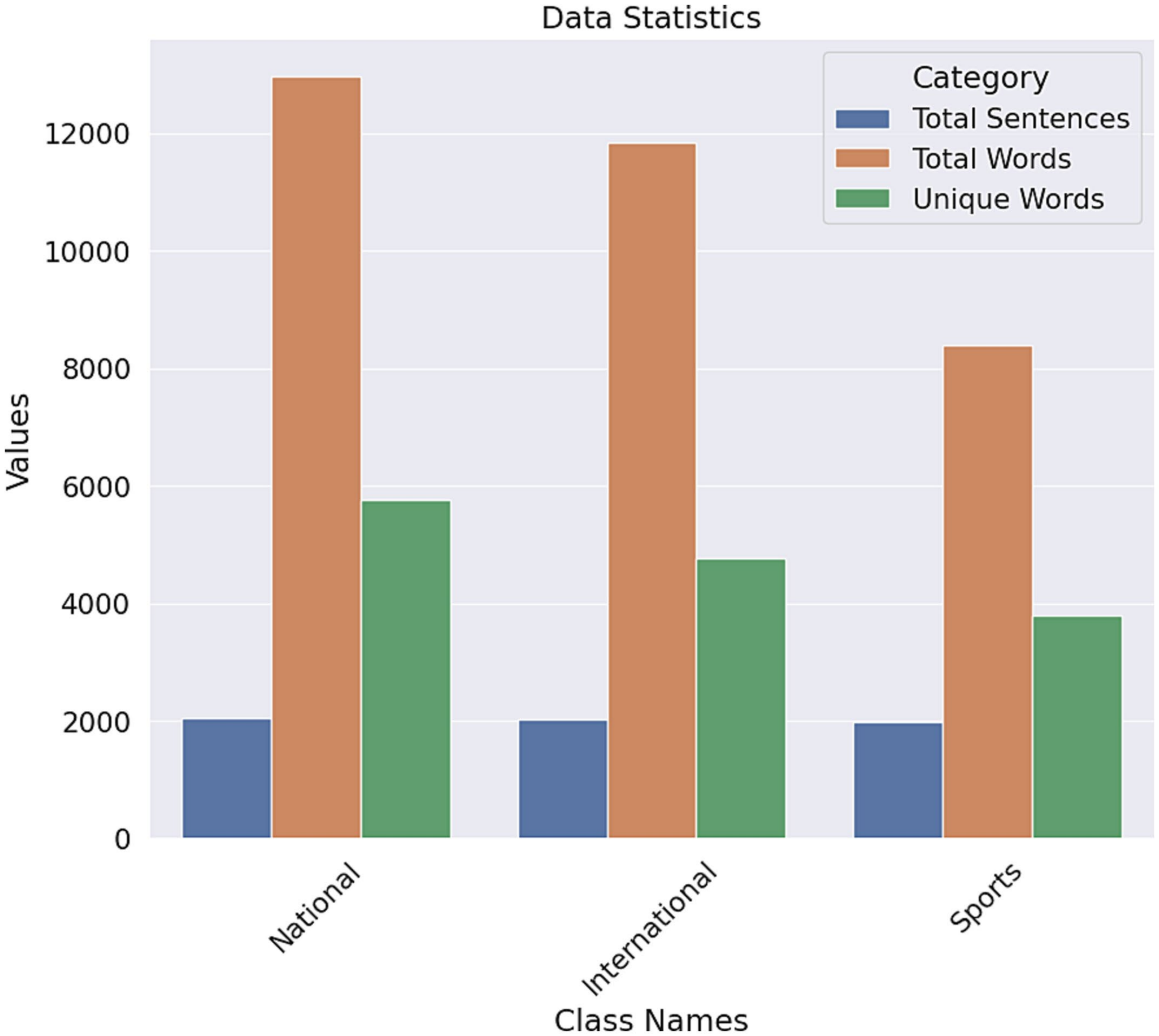
Data statistic bar chart for each class.

The total number of national words is 12,964, and 5,751 of the words are unique. 8,379 words are in the sports category, and 3,788 of the words are unique. In the international category, there are 11,838 words, and 4,765 words are unique. The sports have fewer unique words among the three classes. On the other hand, the national category had the most unique words (Figure 7). There is a simple bar chart of the data statistics for each class.

Table 5 shows the n-gram distributions of the dataset. The frequency of each word in the text is converted into a vector with the help of the TF-IDF. The Ingram range can specify the size of the n-grams. Value 1, 1 shows the unigram where ngrams have a single word, Bigram produces 2 words, and Trigram produces 3 words, which are shown in Table 5.

**Table 5:** N-grams for the news titles based on TF-IDF.

Title	Unigram	Bigram	Trigram
ভারত মহাসাগরে জলদস্যুদের কবলে বাংলাদেশি জাহাজ	(‘রত’, 1)	(‘রত মহ’, 1)	(‘রত মহ গর’, 1)
Bangladeshi ship captured by pirates in	(‘মহ’, 1)	(‘মহ গর’, 1)	(‘মহ গর জলদস’, 1)
Indian Ocean	(‘গর’, 1)	(‘গর জলদস’, 1)	(‘গর জলদস কবল’, 1)

### 3.5 Machine learning

ML algorithms such as Logistic regression, Decision tree, K-nearest neighbors, Random-forest, Support vector machine, Multi. Naive Bayes and Stochastic gradient descent are applied for experimental results on the dataset.

#### 3.5.1 Logistic regression

In supervised learning, Logistic Regression is a statistical way to predict the output based on the given input variables (Cramer, 2002). By using a logistic function, the output maps the values to 0 or 1. The algorithm assumes the output is a linear combination of input variables.

#### 3.5.2 Decision tree

To make a decision, this tree-based algorithm works recursively, whereby the input space is divided into different groups according to the values of features used by each node to construct tree-like decisions for specific attributes (Belson, 1959). One disadvantage of this algorithm is that it overfits noisy data and hence requires pruning strategies.

#### 3.5.3 Random forest

It is a powerful ensemble learning technique that uses a variety of decision trees during the training process. Every tree is trained on some part of the data, and some features are randomly selected. For the classification, the prediction is made by combining outputs from individual trees (Alzubi et al., 2020). The usage of random forests is extensive because they are powerful and can handle large data sets with numerous dimensions (Breiman, 2001).

#### 3.5.4 Multi. Naive Bayes

Multinomial Naive Bayes is a version of the Naive Bayes algorithm especially created for text classification problems in which features are individual words, frequencies, or any other discrete features (Rennie, 2001). In this algorithm, features are independent when it comes to class values. The simplicity of Multinomial Naive Bayes does not make it less effective, as it has been able to work efficiently against many different types of problems. It performs quickly and handles large feature sets too.

#### 3.5.5 K-Nearest neighbors

KNN is an uncomplicated machine-learning algorithm. In the feature space, it ultimately decides the dot class or value by looking at how classes or values are assigned to other data dots that are next to it. It is very simple to understand and can be done easily, but only if the K-neighbours parameter is picked correctly, which states how many neighboring points (K) from each testing sample should be used in making predictions about other classes based on their attributes, along with the kind of measure among them (Cunningham and Delany, 2021).

#### 3.5.6 Support vector machine

The SVM algorithm is useful for carrying out classification tasks. It can establish the ideal hyperplane for separating varied classes by making the margin between support vectors as wide as possible (Boswell, 2002). SVM employs kernel tricks to address non-linearity between classes and features in very many dimensions. For detecting spams and sentiment analysis, this model gives the best output (Qiqieh et al., 2025).

### 3.6 Long short-term memory (LSTM)

The LSTM model used in this study is sequential (Hochreiter, 1997). It processes sequential data into a sequence, and a more abstract representation and gives an output suitable for classification or regression. The embed dim is 64. The input dim is 5,000. The dropout is 0.2, and the recurrent dropout is 0.4. The total parameters in the embedding layer is 320,000. The spatial dropout layer uses 0 input units as a dropout for the embedding layer’s input, which occurs during each training time. The spatial dropout 1D is 0.4. The LSTM is a type of RNN model that is quite popular in Bengali NLP. In this layer, the output shape has been changed into a 64-dimensional vector. There are a total of 33,024 parameters in the LSTM layer. The dense layer is completely linked, and it is activated using softmax. The output shape is (None,3). The total number of parameters in the dense layer is 195. Activation Function (SOFTMAX) converts a real number into a probability distribution. The total number of parameters in the model is 353,219.

### 3.7 Transformer model for classification

Many earlier studies in natural language processing have successfully used transfer learning models like XLM-RoBERTa base, Multilingual BERT, and DistilBERT. We studied these earlier studies and their accuracy on different tasks in detail and resolved to use these models (Hoque et al., 2024 and Roy et al., 2023).

#### 3.7.1 Multilingual BERT

The Bert-based multilingual case is a pre-trained model of the BERT, developed for handling various languages (Kenton and Toutanova, 2019). Masked language modeling is the main goal of this model. This allows the model to understand the languages in the deep contextual meaning.

The base architecture of this model is the 12 transformer layers. Each hidden layer contains 768 neurons. With 12 attention heads in each encoder layer, the self-attention layer focuses on multiple parts of the input. The feed-forward network of each encoder block has 3,072 intermediate sizes. The dropout of attention and the hidden layer are the same, 0.1. The vocabulary size is 119,547. The model can process bidirectional inputs.

This model can fine-tune language tasks by adapting multilingual embeddings to language. The downstream NLP tasks such as sentiment analysis, classification, or NER.

#### 3.7.2 DistilBERT

DistilBERT is a small and faster version of the BERT model on text classification (Sanh, 2019). It takes tokenized text as input and processes it through the DistilBERT encoder, pooling layer, and dense layer. This model has 4 layers of architecture. First is the Input layer, which takes tokenized text as input with a shape of (batch_size, sequence_length). Then, the input text is passed to the encoder layer and processed to generate contextual meaning. Then the pooling layer applies mean pooling to make a sequence-level representation. After that, the dense layer maps the pool and is classified using softmax or sigmoid.

#### 3.7.3 XLM-RoBERTa-base

The XLM-RoBERTa-base model is a transformer-based language model designed for natural language processing (NLP) tasks (Conneau, 2019). It is a lightweight version of the XLM-RoBERTa model, optimized for efficiency while maintaining strong performance. Pre-trained on data in 100 languages, this model is highly versatile for multilingual tasks. Fine-tuning was conducted with a learning rate of 2e-5 times, and the given learning rate was chosen to ensure stability during optimization. The training process spanned five epochs, with a batch size of 16 for both training and evaluation, and input sequences tokenized to a maximum length of 512 tokens. The classification task involved three labels.

The model architecture includes several noteworthy configurations. It employs a dropout probability of 0.1 in the attention mechanism to reduce overfitting, and the GELU activation function is used in the feedforward layers. The hidden layer size is 768, with an intermediate feedforward size of 3,072. The model consists of 12 transformer layers, each with 12 attention heads. A hidden layer dropout probability of 0.1 was also applied. The model supports up to 514 tokens and has a vocabulary size of 250,002, with a total of 278,045,955 trainable parameters. During training, Weights & Biases (W&B) logging was disabled.

The training dataset contained 4,920 examples, processed with a gradient accumulation step of 1, leading to a total of 1,540 optimization steps provided in Equation 1.


$$\mathrm{Optimization}\;\mathrm{Steps}=\frac{Num\;\mathrm{Examples}\times Num\;\mathrm{Epochs}}{\mathrm{Total}\;\mathrm{Train}\;\mathrm{Batch}\;\mathrm{Size}}=\frac{4920\times 5\;}{16}=1540$$ (1)

The evaluation was conducted on a dataset comprising 1,230 examples, also with a batch size of 16. This phase took approximately 33 s, processing 37 samples provided in Equation 2.


Samplespersecond=Stepspersecond×Batch size=2.321×16≈37.1 (2)

The fact that the minimum losses that were recorded in training and the validation stages further indicates that the model has successfully avoided overfitting and that it can easily generalize to the unknown data. As a result, the XLMRoBERTa-base architecture proved to be highly successful and reliable in all the measures that were considered. The construction of the input representation of each of the tokens is defined in Equation 3, where token representations are augmented with positional representations to produce contextualized input representations. Attention mechanism, contextual inter-token relationships, is expressed by means of Equation 4. The nonlinear transformation of the attention outputs, which is done by the feed-forward network, is defined in Equation 5. Introducing layer normalization and residual connections make gradient propagation stable, which is supported in Equation 6. Lastly, the masked language modeling goal objective used in pretraining is elaborated in Equation 7.

Input Representation:


$$x_{i}=E\left(t_{i}\right)+P\left(i\right)$$ (3)

Self-Attention Mechanism:


$$Z^{\left(i\right)}=\mathrm{softmax}(\frac{Q^{\left(l\right)}K^{{\left(i\right)}^{t}}}{\sqrt{d_{k}}}$$ (4)

Feed-Forward Network (FFN):


$$FFN_{\left(z\right)}=\mathrm{ReLU}\left(zW_{1}+b_{1}\right)W_{2}+b_{2}$$ (5)

Layer Normalization and Residual Connection:


$$Xl\left(l+1\right)=\mathrm{LayerNorm}\left(X\left(l\right)+Z\left(l\right)\right)$$ (6)

Masked Language Model Objective:


$$L_{MLM}=\sum_{i\in M}\mathrm{logP}\left(t_{i}|X-M\right)$$ (7)

## 4 Results and discussion

Results and discussion are the key part of the research, which provides a complete perspective on the findings of the research. This part of the paper presents a detailed analysis that has been provided from the dataset, different models’ performance, and evaluation of their scores.

### 4.1 Model evaluation

Based on accuracy, precision, recall, and F1-score, different models are evaluated and compared in their performance. Accuracy, precision, recall, and F1-score are calculated with the formulas given below.

Accuracy: In Equation 8, the accuracy of a model refers to the proportion of predictions it makes, calculated as the ratio of positives and true negatives to all positive and negative observations.


$$\mathrm{Accuracy}=\frac{TP+TN}{TP+TN+FP+FN}$$ (8)

Precision: As expressed in Equation 9, it is made up of the ratio of the number of correct model predictions, which focuses on the precision in relation to positive predictions.


$$\mathrm{Precision}=\frac{TP}{TP+FP}$$ (9)

Recall: From Equation 10, Recall shows how much a given model can identify all instances of a given class correctly, and it is usually calculated as the number of true positives divided by the sum of all numbers that are represented by true positives and false negatives.


$$\mathrm{Recall}=\frac{TP}{TP+FN}$$ (10)

F1 Score: Based on Equation 11, it measure statisticians use when analyzing data. When we combine the two measures (precision and recall), it is called a hybrid measure. The F1-score is calculated as the average of precision and recall.


$$F1=\frac{2*\mathrm{Precision}*\mathrm{Recall}}{\mathrm{Precision}+\mathrm{Recall}}$$ (11)

### 4.2 Comparison of models’ performance

Table 6 shows the comparison of performance by ML models with LSTM. Among the ML models, Multi. Naive Bayes did a good performance with 85.22% accuracy. The best model among the DL and ML models is LSTM, which is the top performer. SVM also performed well, almost matching Naive Bayes. Logistic Regression also delivered a strong performance. However, the other models exhibited some issues. The precision was higher than the accuracy and other metrics. Table 7 shows the performance of BERT models, XLM-Roberta base outperformed with an accuracy of 91.38%. Here, the best two performing models are the XLMRoberta Base and Multilingual BERT, with an accuracy of 91.38 and 87.64%. But the DistilBERT’s performance was poor, infect very poor. Reason is that DistilBERT is trained on English data (Wikipedia + BookCorpus) only. Since, it’s not pre-trained on Bengali data or multilingual data, tokenization mismatched for Bengali text. DistilBERT is not familiar with Bengali vocabulary, this led to not understanding the tokens, thus giving this poor performance.

**Table 6:** Comparison of ML models’ performance.

Models	Accuracy (%)	Precision (%)	Recall (%)	F1-Score (%)
LSTM	86.25	86.25	86.25	86.25
Multi. Naive Bayes	85.22	85.78	85.22	85.16
SVM	84.71	84.98	84.71	84.76
Logistic Regression	81.36	82.26	81.36	81.43
KNN	76.55	77.22	76.55	76.43
SGD	74.00	79.29	74.00	73.79
Random Forest	73.28	79.52	73.28	73.40
Decision Tree	71.56	74.24	71.56	71.76

**Table 7:** Comparison of deep learning and transformer models performance.

Models	Accuracy (%)	Precision (%)	Recall (%)	F1-Score (%)
XLM-Roberta_Base	91.38	91.38	91.41	91.39
Multilingual BERT	87.64	87.79	87.68	87.71
DistilBERT	53.0	52.0	53.0	48.9

From Tables 6, 7, the best-performing model was XLM-Roberta Base, also the 2nd best model is also a transformer-based model. Compared to the ML and DL models, the transformer-based model was quite better, also showing prominent performance.

### 4.3 Confusion matrix comaprison

A confusion matrix is an effective way to evaluate classifier models. From a confusion matrix, a clear vision can be achieved of the outcome of the model and whether the acquired accuracy is valid. Issues such as underfitting or overfitting can also be identified and addressed using a confusion matrix.

Table 8 illustrates the comparative performance of four top-performing models analyzed in this study: XLM-RoBERTa, Multilingual BERT, LSTM, and Multinomial Naive Bayes, respectively. Among these, Multinomial Naive Bayes and LSTM ranked fourth and third, respectively, while Multilingual BERT emerged as the second-best model. The highest-performing model was XLM-RoBERTa, achieving an accuracy of 91.38%. Notably, the BERT-based models demonstrated superior performance compared to both machine learning (ML) and deep learning (DL) models, as evidenced by higher true positive (TP) rates and lower false negative (FN) and false positive (FP) rates. Within the BERT-based models, XLM-RoBERTa outperformed Multilingual BERT, further validating its superior performance in terms of TP and FP metrics.

**Table 8:** Confusion matrix for best-performing ML, DL (LSTM), and Transformer models.

Model name	Class	TP	TN	FP	FN
XLM-RoBERTa BASE	0	334	711	54	65
	1	353	682	65	64
	2	317	775	41	31
Multilingual BERT	0	359	757	66	48
	1	365	749	56	60
	2	354	802	30	44
LSTM	0	334	711	54	65
	1	353	682	65	64
	2	317	775	41	31
Multinominal Naïve Bayes	0	336	709	62	57
	1	316	729	29	90
	2	340	718	81	25

Managing Polysemy and Syntactic Ambiguity: Our BERT-based model tackles polysemous words and syntactic ambiguity in Bengali news headlines using contextual embeddings and self-attention mechanisms. In contrast to static representations, BERT disambiguates words dynamically (e.g., “পদ” as “position” or “foot”) based on bidirectional context analysis. In the case of syntactic ambiguity (e.g., free word order in “দীর্ঘদিনের বৈষম্যের কারণেই সহিংস”), multi-head attention settles dependencies based on weighting pertinent token relationships.

### 4.4 Title classification explanation of XAI-based LIME

#### 4.4.1 Local interpretable model-agnostic explanations (LIME)

We select the ground data coordinates, input them into the black box scheme, and observe the corresponding outputs. This technique assesses the new data based on its proximity to the original coordinate points. Consequently, it fits an alternative model, such as linear regression, to the modified sample set using the derived weights. Henceforth, any original data point can be interpreted using the newly developed explanatory model.

Explainability in a model refers to the capacity to comprehend and interpret the processes by which the model generates its predictions or decisions. While a model may demonstrate high performance and accuracy across various tasks, it often functions as a “black box,” making it challenging to ascertain the rationale behind specific predictions or outcomes. LIME (Ribeiro et al., 2016) initiates the process by altering the Bengali newspaper, introducing subtle modifications such as rearranging, excluding, or inserting words. This approach aims to evaluate the model’s sensitivity to deviations in the input data. The altered Bengali newspaper is then input into the transformer-based black-box model, which produces a prediction. LIME subsequently identifies the key features from the altered instances that are most affected by these modifications. These local surrogate models approximate the behavior of the black-box model in the proximity of the selected instance, providing insight into the underlying decision-making process. The knowledge derived from these local models is then leveraged to explain the predictions made by the black-box model on the original dataset. Typically, these explanations emphasize the terms or critical features that have a significant impact on the model’s decision-making, thereby enhancing the interpretability of the predictive mechanism. Through a systematic and iterative methodology, LIME aids in uncovering the complexities of machine learning models and promotes their transparency, thereby fostering greater confidence in the accuracy of their predictions.

To be more specific, an explanation for a data point x is a model g that minimizes the locality-aware loss L(f, g, πx) associated with how well ‘g’ approximates the original function f in its neighborhood πx while maintaining low complexity, denoted by the model provided in Equation 12.


$$\mathrm{argmi}n_{g}L\left(f,g,\pi_{x}\right)+\Omega \left(g\right)$$ (12)

Table 9 reveals ‘উত’ (weight: −0.0742) as the most significant negative influencer, followed by ‘জন’ (+0.0640) as the strongest positive contributor. Moderate influences include ‘এব’ (−0.0624) and ‘বশ’ (+0.0349), while ‘যৎ’ and ‘ইওয’ show weaker positive effects. These weights indicate each term’s relative importance in the model’s decision-making process, with absolute values quantifying their impact magnitude. These forms of Bengali words are called “ধাতু,” which means verbal root. These words do not carry specific meaning; affixes make them meaningful.

**Table 9:** Weight quantifying their impact magnitude relative importance in the model’s decision-making.

Bengali feature	Raw weight	Absolute weight	Interpretation
উত	−0.0742	0.0742	Strong Negative
জন	+0.0640	0.0640	Strong Positive
এব	−0.0624	0.0624	Moderate Negative
বশ	+0.0349	0.0349	Moderate Positive
যৎ	+0.0277	0.0277	Weak Positive
ইওয	+0.0089	0.0089	Minimal Positive

**Figure 8:**
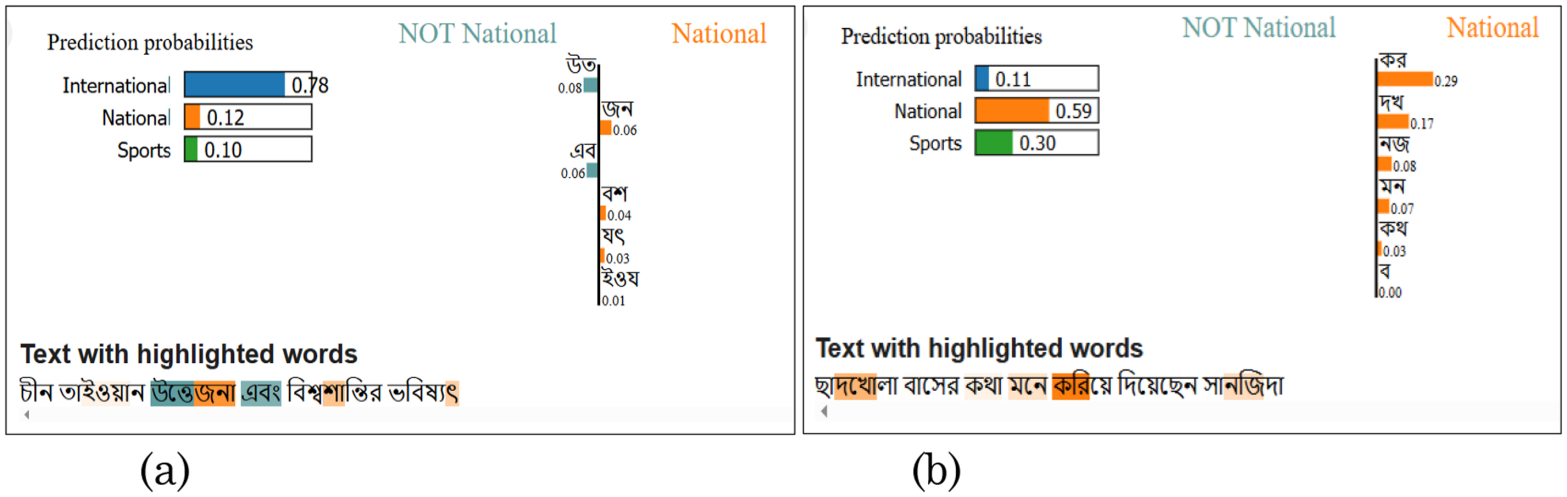
Example XAI-based LIME output for XLM-RoBERTa base for two predictions (misclassified vs. classified). **(a)** Correctly classified **(b)** Misclassified.

In Figures 8a,b, two examples are given for comparing how the model assessed misclassified titles. Figure 8a shows the sentence that has been classified correctly. It says “চীন তাইওয়ান উত্তেজনা এবং বিশ্বশান্তির ভবিষ্যৎ” means “China-Taiwan Tensions and the Future of World Peace” is an international predicted as international as well. The probability score and the weighted value are presented in the figure, which is 0.78, and the weighted values are shown in Table 9. Figure 8b shows the misclassified sentence and how it is assessed. “ছাদখোলা বাসের কথা মনে করিয়ে দিয়েছেন সানজিদা” means “Sanjida reminded me of an open-top bus” misclassified as National instead of Sports. Reason is shown in Figure 8b, that is weight value of each verbal root is positive for national, and the probability for National is 0.59, where the probability for sports is 0.30.

### 4.5 Testing performance with uncertain data

In this subsection, Table 10 shows the predicted output of the proposed model. The actual class is the labeled category of the titles, and the prediction column is the predicted class of the model. It seems that the models performed very well on predicting, though there are very few wrong predictions as well. But still, most of the titles are predicted accurately. It is cross-checked for confusing titles, and model mistakes while predicting.

**Table 10:** Prediction performance of the XLM-RoBERTa model on the test data.

Title	Actual class	Predicted
পাবনার এক খামার উপহার এসেছে ১৯টি চিত্রা হরিণ A farm in Pabna was gifted with 19 Chitra deer	National	National
ইসরায়েলি কর্মকাণ্ড বিবেচনায় আইসিজের সুপারিশ ICJ recommendations regarding Israeli actions	International	International
সত্য ওয়েষ্ট ইন্ডিজকে ছাড়াই বিশ্বকাপ World Cup without Satya West Indies	Sports	Sports
প্লাস্টিকের পুনর্ব্যবহারে নিয়ন্ত্রণ দূষণ Control pollution in plastic recycling	National	National
দীর্ঘদিনের বৈষম্যের কারণেই সহিংস Violence is the result of long-standing discrimination	International	International
মূল অভিযুক্তের বাড়িতে বিক্ষোভকারীদের আগুন Protesters set fire to main accused’s house	National	National
পুরোনো বন্ধু কিসিঞ্জারকে স্বাগত জানালেন জিনপিং Xi Jinping welcomes old friend Kissinger	International	International

### 4.6 Comparison of some similar works

The research gap we found that are shown on the Limitation column of the Table 11. It seems that all the works done in Bengali language were conducted on low amount of data, because of that their models did not perform best. We also found the validation of their models on uncertain data were missing or ignored.

**Table 11:** Comparison with some state of work.

Authors	Contribution	Methods	Accuracy(best)	Limitation
Dhar and Abedin (2021)	News title categorization	Logistic Regression, KNN, Naive Bayes, Adaboost, SVM	73.68% (TF-IDF) 75.78% (Countvect.)	Didn’t validate model’s performance
Das et al. (2021)	Bengali hate speech detection category	1Dconvolutional layers, LSTM, GRU-based decoders	Attention-based decoder 77%	lower accuracy and missing model performance validation
Khan et al. (2021)	Sentiment Analysis	SVM KNN, ANN, Random-forest, Naive Bayes	62%	very poor performance of the models
Our approach	Bengali text classification, news titles categorization	XLM-Roberta	91.38%	

Table 11 compares some works similar to this study based on their dataset, methods, and findings. This comparison provides a clear idea about the gap between the previous works and this study, and finds a better way to classify Bengali news titles.

The result of this study stands out better compared to the papers of some previous studies listed in Table 11. In this study, the average accuracy was 91.38%. The precision, recall value, and F1-score of this research are the same. These previous papers lacked explanations of model performance, and they did not provide any explanation of their model performance validation.

The results of this study represent an optimized outcome, demonstrating superior performance compared to other works in the field. Many of the reviewed studies reported lower accuracy scores and exhibited minimal differences between accuracy and other performance metrics. In some cases, the results were notably suboptimal, with very low accuracy, potentially due to inadequate preprocessing of the dataset. In this study, the integration of LIME played a pivotal role in evaluating the models’ understanding of the provided dataset. This approach not only ensured robust model performance but also enhanced interpretability. The outcomes of the proposed model are noteworthy and underline its effectiveness in addressing the research problem.

## 5 Conclusion and future scope

This research focuses on Bengali news article classification employing various machine learning models and deep learning techniques, emphasizing LSTM neural networks and BERT base models. We aimed to improve the classification precision of predefined categories of Bengali news articles to aid future growth in the NLP domain, specifically for low-resource languages such as Bengali. This research emphasizes the effectiveness of deep learning models on text classification tasks, specifically in Bengali. It points out the interpretability of Explainable AI (XAI), ensuring that the methods we apply are not only functional but also well understood by all people. We aim to encourage more research and innovation in the field of NLP, focusing on low-resource languages such as Bengali, by proposing a method of categorizing Bengali news articles and solving its unique issues.

For future works, we will increase our dataset to improve model accuracy, perform fine-tuning and hyperparameter optimization to improve the model’s performance, and also integrate additional explainability techniques to further improve model clarity.

### 5.1 Experimental setup

Experiments were conducted with a combination of local and cloud computing resources. We worked on a system powered by a 12th Gen Intel(R) Core(TM) i5-12450H processor at 2.00 GHz with 16 GB of RAM (15.7 GB usable), on a 64-bit Windows operating system with an x64-based processor architecture. We utilized Google Colab to train and fine-tune the model on the NVIDIA T4 GPU, which is easily accessible within the Colab environment. Training was completed in a total of around 38 min and 55 s, with throughput equal to 10.534 training samples/s and 0.659 training steps/s.

## Data Availability

The datasets presented in this study can be found in online repositories. The Dataset is publicly available on Mendeley. (doi: 10.17632/g6ygmy7s5r.2).
